# Editorial: Rising stars in exercise physiology

**DOI:** 10.3389/fphys.2023.1177745

**Published:** 2023-03-09

**Authors:** David C. Andrade, Luca P. Ardigò, Céline Aguer, Anthony S. Leicht

**Affiliations:** ^1^ Exercise Applied Physiology Laboratory, Centro de Investigación en Fisiología y Medicina de Altura (FIMEDALT), Departamento Biomédico, Facultad de Ciencias de la Salud, Universidad de Antofagasta, Antofagasta, Chile; ^2^ Department of Teacher Education, NLA University College, Oslo, Norway; ^3^ Faculty of Medicine and Health Sciences, McGill University Campus Outaouais, Gatineau, QC, Canada; ^4^ Institut du Savoir Montfort—recherche, Ottawa, ON, Canada; ^5^ Department of Biochemistry, Microbiology and Immunology, Faculty of Medicine, University of Ottawa, Ottawa, ON, Canada; ^6^ Sport and Exercise Science, James Cook University, Townsville, QLD, Australia; ^7^ Australian Institute of Tropical Health & Medicine, James Cook University, Townsville, QLD, Australia

**Keywords:** heart rate, cardiac autonomic modulation, metabolic control, muscle function, exercise

Physical exercise has been recognized as essential for human health and evolution for thousands of years, beginning with the ancient cultures. Hippocrates, Plato, Aristotle, and the Roman physician Galen were the earliest recorded and most well-known promoters of the beneficial effects of physical exercise. Since these times, several dedicated laboratories worldwide have been established, with many researchers conducting numerous investigations related to exercise physiology; nevertheless, a cornerstone of all laboratories is the development of new and novel researchers. These talented and emerging researchers have been necessary for our understanding of exercise physiology to have reached where we are today (and where we will be in the future). Given the evolution of exercise physiology, the field has incorporated a range of basic to applied scientific investigations and a range of end-users (e.g., researchers, athletes, coaches, physiologists, and clinical/public health professionals) who will benefit from these new advances in exercise physiology.

Accordingly, we were delighted to develop a Research Topic called “*Rising Stars in Exercise Physiology*” to showcase the work of these emerging researchers. This compilation of original research, reviews, and minireviews illustrates the excellent work of internationally recognized researchers in the early stages of their independent careers. This Research Topic focuses on autonomic and chemoreflex control following acute and chronic exercise training, glucose control during exercise, and muscle function/adaptations.

Firstly, Arce-Álvarez et al. reviewed the possible role of peripheral and central chemoreceptors as pivotal sensors for the breath-hold duration in swimming athletes. These authors proposed that swimmers display a decreased chemoreflex control, which confers resistance to an apnea event at rest and during exercise (dynamic apnea). Adding to the topic of breath-hold-related hypoxia, high altitude was considered a type of hypoxia for physiological adaptations in the work of Cerda-Kohler et al.. These authors showed that 3 weeks of training at a moderate altitude (2,900 m above sea level) compared to the “live high train high” paradigm was able to improve breathing response to exercise at sea level in elite rowers. Extending our theme of autonomic control, Castillo-Aguilar et al. focused on cardiac autonomic modulation as a mechanism for muscular fatigue in young swimmers following high-intensity exercise over two consecutive competition periods. They reported that males displayed greater parasympathetic reactivity after the anaerobic muscular fatigue (Wingate) test across the competition periods. Conversely, females exhibited lower cardiac autonomic modulation following the Wingate test during the same competition periods.

While exercise physiology is central to our understanding of athletic performance, it also contributes to our understanding of pathophysiological conditions. For example, Nelson et al. assessed heart rate (HR) variables in patients with myalgic encephalomyelitis/chronic fatigue syndrome (ME/CFS) who completed two progressive maximal cardiopulmonary exercise tests (CPETs) on consecutive days. These authors reported that patients with ME/CFS displayed higher ventilation during the second CPET. Further HR at the ventilatory threshold was greater after the second CPET, and HR recovery was revealed to be an inadequate sensitive and specific biomarker of the fatigued condition.

The second theme for our Research Topic was glucose/metabolic function and exercise training. Alvarez et al. compared the residual effect (post-24 h and post-72 h) of three different training modalities of 12-week-long resistance training (RT), high-intensity interval training (HIIT), and concurrent training (combination of RT and HIIT) on fasting plasma glucose and insulin in insulin-resistant women. They demonstrated that HIIT decreased fasting plasma glucose, while RT decreased fasting insulin at 24 h post-training. They also showed that at 72 h post-training, HIIT and RT had better residual effects than the concurrent training regime on fasting plasma glucose. By testing different regression models, they showed that the reduction in adiposity and increase in skeletal muscle mass and bone better explained the poorer residual effects promoted by concurrent training.

Continuing this metabolic theme, Delgado-Floody et al. determined the non-responder rate after a 20-week program of RT and HIIT in two groups of women with morbid obesity (i.e., a high metabolic syndrome risk group and a low metabolic syndrome risk group). The exercise training intervention was more efficient overall in the group with a high metabolic syndrome risk, which showed improved blood glucose levels and other metabolic risk factors. However, the high metabolic syndrome risk group also showed a higher non-responder prevalence for improving different metabolic syndrome factors.

The final theme of our Research Topic concentrated on exercise training and muscular function/adaptations. Akbar et al. systematically reviewed the impact of neuromuscular training (i.e., combined sport-specific and fundamental movements) on physical fitness components in athletes. From 18 studies, training was primarily conducted *via* plyometric and strength exercises, with positive effects reported for muscular endurance and power, speed, agility, balance, and coordination amongst a range of athletes. Practical recommendations for pre- and in-season training were provided along with a call to further examine the influence of neuromuscular training on other fitness components and sporting populations. To close, Beaudry et al. reviewed the role of dietary protein in altering myogenic muscle stem cell and fibro-adipogenic progenitor responses to endurance and resistance exercise in healthy and clinical populations. The authors concluded that protein supplementation with resistance exercise increased myogenic muscle stem cell content/activity. In contrast, resistance exercise alone increased the number of fibro-adipogenic progenitor cells (i.e., no additional impact of protein supplementation) in healthy populations. Moreover, protein supplementation provided no additional benefits to resistance exercise for clinical populations. For endurance exercise, protein supplementation increased the initial myogenic response to an acute bout but did not increase it following chronic training. Further studies examining the influence of diet and exercise on myogenic cell content were recommended to improve muscular health for healthy and clinical populations.

The current Research Topic has highlighted the work of “rising stars” within the exercise physiology field who were nominated by their peers. The continued support for these and other emerging researchers will ensure that the future of exercise physiology is advanced for improved performance and health. We look forward to the future advancement of the exercise physiology field.

